# Verification of the Chromosome Region 9q21 Association with Pelvic Organ Prolapse Using RegulomeDB Annotations

**DOI:** 10.1155/2015/837904

**Published:** 2015-08-10

**Authors:** Maryam B. Khadzhieva, Dmitry S. Kolobkov, Svetlana V. Kamoeva, Anastasia V. Ivanova, Serikbay K. Abilev, Lyubov E. Salnikova

**Affiliations:** ^1^Laboratory of Ecological Genetics, N.I. Vavilov Institute of General Genetics, Russian Academy of Sciences, 3 Gubkin Street, Moscow 117971, Russia; ^2^The Faculty of Bioengineering and Bioinformatics, Lomonosov Moscow State University (MSU), GSP-1, Leninskiye Gory, 1-73, Office 433, Moscow 119991, Russia; ^3^Laboratory of Animal Genetics, N.I. Vavilov Institute of General Genetics, Russian Academy of Sciences, 3 Gubkin Street, Moscow 117971, Russia; ^4^Department of Obstetrics and Gynecology, Pirogov Russian National Research Medical University, 1 Ostrovitianov Street, Moscow 117997, Russia; ^5^Laboratory of Molecular Immunology, Federal Research Center of Pediatric Hematology, Oncology and Immunology Named after Dmitry Rogachev, the Russian Ministry of Health and Social Development, 1 Samora Machel Street, Moscow 117198, Russia

## Abstract

Pelvic organ prolapse (POP) is a common highly disabling disorder with a large hereditary component. It is characterized by a loss of pelvic floor support that leads to the herniation of the uterus in or outside the vagina. Genome-wide linkage studies have shown an evidence of POP association with the region 9q21 and six other loci in European pedigrees. The aim of our study was to test the above associations in a case-control study in Russian population. Twelve SNPs including SNPs cited in the above studies and those selected using the RegulomeDB annotations for the region 9q21 were genotyped in 210 patients with POP (stages III-IV) and 292 controls with no even minimal POP. Genotyping was performed using the polymerase chain reaction with confronting two-pair primers (PCR–CTPP). Association analyses were conducted for individual SNPs, 9q21 haplotypes, and SNP-SNP interactions. SNP rs12237222 with the highest RegulomeDB score 1a appeared to be the key SNP in haplotypes associated with POP. Other RegulomeDB Category 1 SNPs, rs12551710 and rs2236479 (scores 1d and 1f, resp.), exhibited epistatic effects. In this study, we verified the region 9q21 association with POP in Russians, using RegulomeDB annotations.

## 1. Introduction

Pelvic organ prolapse (POP) is the dropping of the pelvic organs caused by weakness or damage to the normal support of the pelvic floor. Prevalence of a disease state (stage II prolapse or greater) varies by data source from 3% of parous women [1] to 19% of women with advanced disease requiring surgery [2]. As many as 50% of women may have some degree of POP. Clinical manifestations related to POP often become evident after menopause [3], though it is becoming a serious health problem for women of all ages with first symptoms that might be experienced before age 30 [4]. POP rarely occurs as a separate condition and often correlates with urinary and faecal incontinence, sexual dysfunction, psychological, and social disadaptation [5]. Risk factors, which predispose to POP, include pelvic floor injury (vaginal parity and pelvic floor trauma during childbirth), lifestyle and health conditions (mainly, menopause, body mass index (BMI), chronic cough, constipation, and heavy lifting), genetic-related conditions (ethnicity, connective tissue disorders, and familial character of prolapse) [6, 7]. Forty-three percent of the variation in susceptibility for POP may be explained by genetic effects [8].

The majority of multifactorial disorders are characterized by a large spectrum of genetic variations in disease modifying genes, whereas information about causative polymorphic genes is scarce. In contrast, the genetic studies of POP have been mainly focused on a limited number of causative genes, among them are the genes controlling the collagen and elastin synthesis and remodeling [9–12], extracellular matrix metabolism [9, 13–16], and hormone receptors [17–19]. There are also three genome-wide linkage studies of the same group of researchers that have determined chromosome 9q21 [20], six other loci [21], and chromosomes 10q24–26 and 17q25 [22] as the regions associated with a predisposition for advanced POP in European pedigrees. It is known that family-based genetic studies may be unsuccessful for complex traits in general population [23] and the results should be validated in independent studies. No candidate gene studies have been performed yet from these genetic regions.

Functional SNPs in a specified chromosome region can be chosen with a powerful tool, RegulomeDB, a database which provides functional annotations of SNPs in the human genome using data sets from ENCODE and other sources [24]. These annotations include data on chromatin structure, methylation, protein motifs, and binding. RegulomeDB presents a scoring system, with categories ranging from 1 to 7, where category 7 variants lack evidence of regulatory function, while category 1 variants are those “likely to affect binding and linked to expression of a gene target” [24]. Category 1 is further divided into 1a–1f subcategories. A variant scored as 1a has the highest confidence on functionality. To date, functional annotation data have been mostly used for a* post hoc* analysis of GWAS data [25–28] (Hong et al., 2014; Rajkumar et al., 2014; Rosenthal et al., 2014; Zia et al., 2015) while selection of putative functional SNPs may also be useful in future studies.

To verify GWAS findings on POP association with the 9q21 chromosome region [20] and six other loci (rs1455311 (4q21), rs1036819 (8q24), rs430794 (9q22), rs8027714 (15q11), rs1810636 (20p13), and rs2236479 (21q22)) [21], we conducted an association study, in a Russian population, between POP risk and twelve SNPs that have been identified in the above studies or selected with RegulomeDB annotations for the region 9q21.

## 2. Materials and Methods

### 2.1. Subjects

The study was approved by the Ethics Committee of the Pirogov Russian National Research Medical University (university review board approval number 117 (April 16, 2012)) and was conducted according to the principles of the Declaration of Helsinki; all patients provided written informed consent.

Based on the POP-Q examination, POP patients were diagnosed with stages III-IV prolapse and controls without prolapse and prior history of prolapse surgery (stage 0). Subjects were recruited from the clinical bases of the Department of Obstetrics and Gynecology of the Pirogov Russian National Research Medical University (RNRMU), Moscow, Russia (from December 2011 to September 2013). Exclusion criteria for the group under study included family history of pelvic organ prolapse, lack of informed consent, pregnancy, gynecologic malignancy, and hereditary (Marfan or Ehlers-Danlos syndromes) or acquired connective tissue disorders (rheumatoid arthritis, systemic lupus erythematosus, scleroderma, polymyositis, and dermatomyositis). We also carefully collected data on perineal trauma and episiotomy (surgical incision, which is made to prevent more extensive vaginal traumas during childbirth). These data were collected based on the copies of the hospital medical records and the results of physical examination (the scars). Women with perineal trauma and episiotomy were combined in the group “perineal trauma in childbirth,” since both conditions lead to pelvic muscle weakness, higher risk of third- and fourth-degree traumas, and pelvic floor disorders [29].

To compare the studied genotypes distribution in Russian population and HapMap populations, we included the following populations from the project [30]: Caucasian populations, Utah residents with Northern and Western European ancestry (СEU), and Toscans in Italy (TSI); Asian populations, Chinese in Metropolitan Denver, Colorado (CHD), and Japanese in Tokyo (JPT); African populations, Yoruba in Ibadan, Nigeria (YRI), and Luhya in Webuye, Kenya (LWK); and two other populations, Gujarati Indians in Houston, Texas (GIH), and Mexican ancestry in Los Angeles, California (MEX). SNP genotype data were downloaded from the HapMap project site (The International HapMap Consortium, 2003), all HapMap samples were divided by gender, and only female samples (CEU, *n* = 89; TSI, *n* = 44; CHD, *n* = 44; JPT, *n* = 45; YRI, *n* = 80; LWK, *n* = 45; GIH, *n* = 43; MEX, *n* = 43) were included in a principal component analysis.

### 2.2. SNPs Selection and Genotyping

The purpose of this study was to replicate the results of genome-wide linkage and genome-wide association studies for pelvic organ prolapse. In the linkage study of high-risk POP pedigrees, 9q21 region (between rs4077632 and rs10868525 (Assembly GRCh37.p13, 9: 81163473–89618028 bp)) has shown an evidence for a predisposition to POP [20]. The top effect was found for rs11139451. Some other intergenic markers (e.g., rs4077632) and possible genes candidates in the region (e.g.,* TLE1* and* TLE4*) have been mentioned. Additionally to the SNPs cited by the authors (rs4077632 and rs11139451), we chose four markers utilizing RegulomeDB functional annotations for the region 9q21. There are SNPs rs2777781 in the gene* TLE1* (RegulomeDB score 3a), rs2807303 in the gene* TLE4* (RegulomeDB score 2a), and rs12237222 (RegulomeDB score 1a) and rs12551710 in the gene* FRMD3* (RegulomeDB score 1d). Six other SNPs have been associated with POP in the more recent genome-wide association study of Allen-Brady et al. [21], that is, rs1455311 (4q21), rs1036819 (8q24), rs430794 (9q22), rs8027714 (15q11), rs1810636 (20p13), and rs2236479 (21q22). These SNPs were also included in our investigation.

DNA was isolated from 200 *μ*L of blood using gDNA purification kit Diatom DNA Prep 200 (Isogene laboratory, Moscow, Russia). The genotyping was performed with a PCR-CTPP (polymerase chain reaction with confronting two-pair primers) [31]. Amplification was carried out using an ABI thermal cycler with two external and two internal sequence-specific primers (Supplementary Table 1, in Supplementary Material available online at http://dx.doi.org/10.1155/2015/837904) and tubes PCR MasterMix (Isogene laboratory, Moscow, Russia). The PCR products were analyzed in ethidium bromide-stained 2% agarose gel. Blinded duplicates (10%) of randomly taken DNA samples for each SNP were genotyped once more with 100% concordance in genotype calling.

### 2.3. Statistical Analysis

Categorical demographic and clinical variables were assessed using Fisher exact test (two-tailed). Since continuous demographic and clinical variables did not assume a normal distribution, the Mann-Whitney *U* nonparametric test was used to compare such variables. Deviation from Hardy-Weinberg equilibrium was assessed by *χ* ^2^ analysis. Logistic regression analysis implemented in SNPStats package [32] was performed to assess associations between studied SNPs and POP. In multivariate analysis, we adjusted adjusted for main covariates: age, BMI, vaginal parity, and vaginal trauma in childbirth. Genetic model was selected using the Akaike Information Criterion (AIC) value with the lowest AIC value considered corresponding to the best-fitting model for the fitted variant. For genotypes with minor allele frequencies <5% only dominant and additive genetic models were evaluated. A two-tailed *P* value < 0.05 was considered statistically significant.

Pairwise linkage disequilibrium (LD) values were measured as Lewontin's *D*′-values and estimated from genotype data using the expectation-maximization algorithm implemented in SNPStats software. The SNPStats software was also used to evaluate haplotype association with POP by individual haplotype *P* values.

Gene-gene interactions were estimated using “SNPassoc” package [33] within R statistical software [34] performing log-likelihood ratio tests (LRTs).

WINPEPI test power calculator [35] was used to assess sample size and test power. Assuming that odds ratio (OR) ≥ 2.0 is clinically meaningful [36], we established test power in the range of 70.30% to 96.06% (at two-sided significance level) for this effect size, sample size *n* = 500 (300 controls and 200 cases), and risk genotype frequencies in a range of 10%–50%.

Successful replication study presents a statistically significant association of the same genetic variant in the same direction [37]. Our study cannot be considered a classic replication study since prior results have been obtained in a genome-wide linkage analysis for a gene region. Nevertheless, our results provide verification of the association of pelvic organ prolapse with the 9q21 haplotype in Caucasians. It is accepted that replication studies do not require genome-wide multiple testing correction [38]. In our study, we also did not adjust for multiple comparisons since we tested robust statistical associations [39, 40]. The Bonferroni correction was applied when testing for SNP interaction.

To detect admixture, we investigated Russian and HapMap populations structure via the discriminant analysis of principal components (DAPC) [41]. It was evaluated using the adegenet package in R statistical software [42].

Interpopulation differences in allele frequencies were assessed with pairwise Weir and Cockerham's Fst estimator (Fst), using package diversity [43].

## 3. Results

### 3.1. SNPs Characteristics

SNPs considered in the study are described in Table 1. Of the 12 studied SNPs, five SNPs are intergenic, while seven SNPs are located in the genes. Among these seven SNPs, five SNPs are related to the protein-coding genes,* TLE1* (rs2777781),* TLE4* (rs2807303),* COL18A1* (rs2236479),* ZFAT* (rs1036819), and* FRMD3* (rs12551710), and two SNPs are mapped to the RNA gene* LINC01088* (rs1455311) and uncharacterized gene* LOC102723989* (rs12237222). With few exceptions (rs2807303 and rs1036819), the majority of the SNPs assigned to the genes lie within intron regions. The choice of the genetic markers rs12237222 (score 1a) and rs12551710 (score 1d) in the region 9q21 was based on the RegulomeDB annotations. We present here the comparative qualitative and quantitative characteristics of the RegulomeDB SNPs in the categories 1a–1f in the whole human genome and in the region 9q21 (Table 2). Among the relatively small number of the SNPs scored 1a in the whole human genome, only one SNP with a score 1a was found in the region 9q21. This is the SNP rs12237222.

### 3.2. Characteristics of the Study Population

In total, 210 patients with pelvic organ prolapse (stages III-IV) and 292 control subjects with no even minimal prolapse were included in the study. The description of the study population is presented in Supplementary Table 2. Briefly, our cases and controls were similar in ethnicity (Caucasians from the European region of Russian Federation) and age (57.65 ± 10.80 and 57.25 ± 12.70, resp.). Higher BMI (29.17 ± 5.85 and 27.46 ± 6.56, *P* = 5.6 × 10^−4^), vaginal parity (1.69 ± 0.65 and 1.46 ± 0.54, *P* = 0.014), and perineal trauma in childbirth (52.45% versus 32.75%, *P* = 1.7 × 10^−5^) were found in POP patients than in matched controls.

### 3.3. Single-Locus Analysis

All SNPs were in Hardy-Weinberg equilibrium in the cases and in the controls. In the crude analysis, the minor rs12237222-G allele frequency was found to be higher in the cases than in the controls under the dominant model (Table 3). This effect disappeared in the multivariate analysis. Other SNPs were not associated with POP neither in the crude nor in the multivariate analysis (Supplementary Table 3).

### 3.4. Haplotype Analysis

For the haplotype analysis, we included all studied SNPs located in the region 9q21 (Table 4). Haplotype number four comprising rs12237222-G allele was significantly associated with POP in the analysis adjusted for age, BMI, vaginal parity, and peritoneal trauma in childbirth. When we excluded this SNP from the haplotype analysis, we found that haplotype number one that included risk alleles from the remaining five SNPs became the reference haplotype (the most common). The pronounced effect was also found for the two-SNP-haplotype rs2777781-A/rs12237222-G with total haplotype frequency 0.3087 (Table 4). No significant association was observed between other haplotypes and POP development.

It is interesting to note that rs12237222 is in weak but highly significant linkage disequilibrium with rs11139451 (*D*′ = 0.257, *P* = 1.45 × 10^−6^) that has been found to show the maximum HLOD score in the study of Allen-Brady et al. [20] (Supplementary Table 4).

### 3.5. Analysis of SNP Interaction

**Figure 1:**
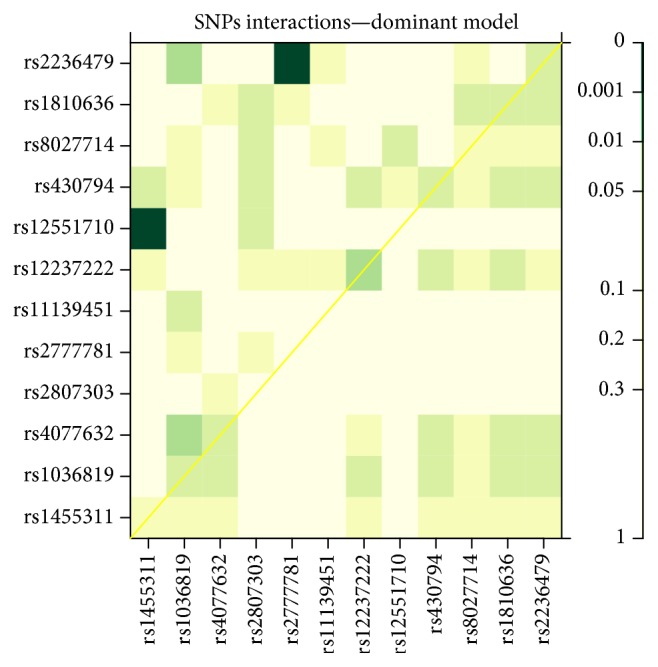
Gene-gene interaction plot. The plot shows the significance levels of gene-gene interactions and pelvic organ prolapse (POP) in the dominant model. Each plot indicated the *P* values preceded by different likelihood ratio tests. Different colors show different levels of statistical significance. The diagonal line contains the *P* values from likelihood ratio test for the crude effect of each SNP. The upper triangle in the matrix presents the *P* values for the interaction (epistasis) log-likelihood ratio test. The lower triangle shows the *P* values from likelihood ratio test comparing the two-SNP additive likelihood to the best of the single-SNP models.

Figure 1 presents a heat plot color map for an analysis of SNP interaction. Cells are colored according to interaction *P* value (less significant effects, light yellow; more significant effects, green). The most significant interactions (dark green cells) were observed for two SNP pairs, rs12551710 (RegulomeDB score 1d) and rs1455311 (RegulomeDB score 5) (*P* = 7.3 × 10^−5^), from first interaction pair and rs2236479 (RegulomeDB score 1f) and rs2777781 (RegulomeDB score 3a) (*P* = 1.7 × 10^−4^) from second interaction pair. It should be highlighted that these highly significant results did not reflect statistical interaction, since the above SNPs were not associated with POP in our single-locus analysis. Given that interaction results were novel, we used correction for multiplicity considering all possible combinations (12 × 12 = 144). The results remained significant after Bonferroni correction for twelve SNP pairs (*P* = 0.011 and *P* = 0.024, resp.).

### 3.6. Analysis of Population Structure

**Figure 2:**
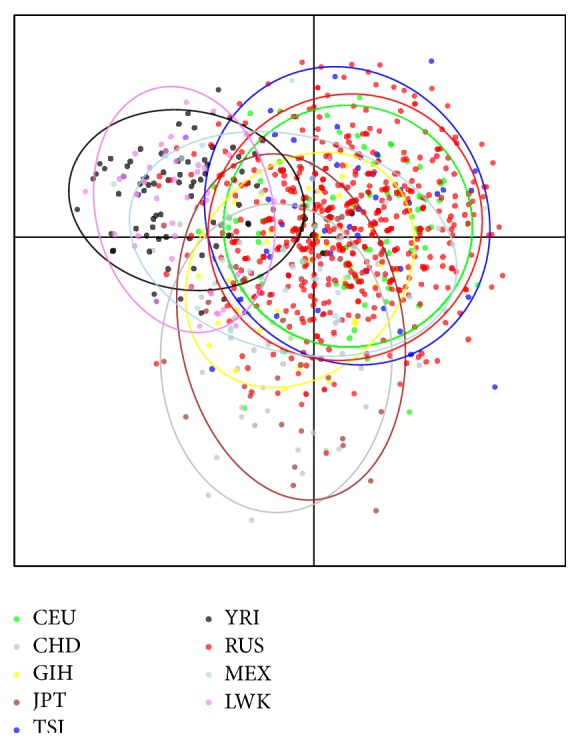
Structure analysis of 12 SNPs in this data (RUS) and in other Caucasian populations (CEU, Utah residents from Northern and Western European and TSI, Toscans from Italy), Asian populations (CHD, Chinese from Metropolitan Denver, Colorado, and JPT, Japanese from Tokyo), African populations (YRI, Yoruba from Ibadan, Nigeria, and LWK, Luhya from Webuye, Kenya), and two other populations (GIH, Gujarati Indians from Houston, Texas, and MEX, Mexican ancestry in Los Angeles, California). Only female samples were included in a principal component analysis. HapMap data were absent in TSI, MEX, GIH, CHD, and LWK populations for rs1223722 and rs12551710 and in CHD population for rs8027714.

The discriminant analysis of principal components showed that Russian population has no internal stratification (Figure 2). Russian population was located close to other Caucasian populations, CEU and TSI. African YRI and LWK populations and Asian CHD and JPT populations were far from Caucasian populations, while GIH and MEX populations took an intermediate position (Figure 2).

Fst statistics are presented in Supplementary Table 5. The pairwise Fst values between the examined populations were in line with the data of DAPC. The lowest pairwise Fst values (Fst = 0) were revealed for CEU-RUS and CEU-TSI populations.

## 4. Discussion 

In this gene candidate study, we tested the association of the region 9q21 and six other loci [20, 21] with advanced POP in Caucasians. We used RegulomeDB resource to select regulatory SNPs in the region 9q21. There was only one SNP rs12237222 in the region with the best RegulomeDB score 1a. This SNP appeared to be the key SNP for the 9q21 haplotype associated with POP. Two other category 1 variants, which are also characterized as eQTL, rs2236479 (21q22), and rs12551710 (9q21) exhibited epistatic (nonlinear interaction) effects with rs2777781 and rs1455311, respectively. To control for the confounding effect of known clinical covariates (age, BMI, vaginal parity, and pelvic floor trauma in childbirth), we adjusted for these factors. To assess hidden population stratification, we compared the genotypic frequencies of 12 studied SNPs to those of HapMap ethnic groups and found that our sample showed little structure.

In terms of ancestry, Russians are Caucasians. The study of Russian population was representative, from an ethnic standpoint of the other Caucasian populations (CEU and TSI). Other HapMap populations in this study, YRI, LWK, JPT, and CHD populations differed significantly from all three Caucasian populations, Russian, CEU, and TSI. It is worth noting that RUS, CEI, and TSI populations and relatively close to them GIH and MEX populations are characterized by high prevalence of POP [4, 44, 45]. In contrast, African and Asian women presented in our study by YRI, LWK, JPT, and CHD populations are less prone to POP development [4, 44, 46, 47]. This may be explained mainly by anatomical features [48, 49]. Nevertheless, it is an interesting challenge for future research directions to determine whether ethnic-specific genotype frequencies of the studied SNPs make any contribution to higher prevalence of POP in Caucasian, GIH, and MEX females compared with Asian and African females.

All our patients had an advanced prolapse (stages III-IV on the POP-Q examination), while our controls had not even minimal prolapse. Consideration of more extreme phenotypes is recommended for the genetic association studies [50]. Utilizing extreme phenotypes increases the association effects, with improved statistical power of relatively modest sample sizes [51]. In relation to POP, this direction could be taken into account not only for patients but for controls as well. In a recent study of Wu et al. [15], their control group included controls with no or minimal prolapse, who were older than patients. We applied another approach for recruitment of an appropriate comparison group. Should POP arise, we would expect that subclinical manifestation progresses to more severe symptoms. The rate of this progress depends on many variables including the history of vaginal childbirth and other clinical conditions (e.g., hysterectomy), age, frequent heavy lifting, and intense physical activity leading to the repetitive increase of pelvic pressure [52]. For this reason, we purposely included women without any subclinical POP in our control group.

It is known that over 98% of the human genome comprises noncoding DNA. However, the Encyclopedia of DNA elements (ENCODE) project has recently demonstrated that a biochemical function could be assigned to 80% of the human genome [53]. Regulatory activity of noncoding DNA explains the fact that a very high amount (90%) of GWAS-identified SNPs associated with multifactorial diseases and complex traits are located in noncoding regions [54]. RegulomeDB includes data sets from ENCODE and other sources to predict regulatory function of a SNP in a score based system. Lower scores mean increasing evidence that a variant is located in a functional region: category 1 indicates that a variant has the highest likelihood of being linked to the expression of a target gene and category 1a is characterized by a full set of features (eQTL + TF binding + matched TF motif + matched DNase Footprint + DNase peak). RegulomeDB project provides an important tool for both tasks, planning an association study and* post hoc* analysis; its data are useful for generating hypotheses and explaining possible epistasis interactions.

SNP interaction might be common among eQTL SNPs that influence gene expression. Epistatic interactions involve local or distant factors acting in* cis* or* trans*.* Cis*-interaction usually occurs within 500 kb region and may sometimes reflect an underlying haplotype effect, while transinteraction indicates interaction between SNPs from distant regions [55]. Loci involved in gene-gene interactions may not show associations on their own. It is also known that in replication studies some loci associations could not be verified, but the corresponding regions might show clear interaction signals [56]. We could not replicate all associations from the studies of Allen-Brady et al. [20, 21], but the interaction effects revealed in our study for several SNPs are in line with the associations found in the above studies for the relevant SNPs.

It is proposed that epistasis among regulatory variants can have strong phenotypic effects, since gene regulation has an important role in adaptation to different harmful stimuli. Regulatory SNPs often lie in noncoding sequences and affect transcription of coding and noncoding RNAs or RNA functions and processing [57]. In our study, among SNPs associated with POP in the haplotype and/or interaction analysis, three SNPs (*TLE1* rs2777781,* FRMD3* rs12551710, and* COL18A1* rs2236479) were located within the genes with possible impact upon development and organization of muscle and connective tissue of the pelvic floor [20, 21]. Their effects may be realized based on regulation of the expression of these genes or via transregulatory activity. Two other SNPs, rs1455311 and rs12237222 (the top SNP in this assay), within the nonprotein coding genes were also associated with POP in the haplotype or interaction analysis. Based on data of SCAN SNP and CNV Annotation Database [58] assayed in lymphoblastoid cells, these SNPs appeared to exhibit transregulatory activity in CEU population: rs1455311 was associated with the expression of the* NAPRT1* (nicotinate phosphoribosyl transferase domain containing 1) gene and rs12237222 influenced the expression of the* CAT* (catalase) and* GYPE* (glycophorin E) genes.* NAPRT1* and* CAT* genes encode antioxidant enzymes and control response to oxidative stress, which is known to play a role in the development of many chronic or late-onset diseases including POP [59]. Regulatory SNPs may exert a tissue-dependent effect on gene expression but in general, “allele specific expression differences between individuals dominate over tissue-specific effects” [60]. Basing on these data, we can speculate that the above SNPs may be associated with POP in haplotype or interaction dependent mode, in particular through influence on efficacy of oxidative stress response.

A limitation of our study is that, firstly, our sample size was modest and the study is powered to detect only relatively large effect sizes (minimum detectable OR~1.7). Secondly, the study is limited to the population of Russian Federation. The strength of the study is in the sampling of extreme phenotypes, the inclusion of all main risk factors for POP in multivariate regression analysis, little population structure, and using of RegulomeDB resource to select functional SNPs.

## 5. Conclusions

The findings of the present study verified the results of the genome-wide linkage analysis in European pedigrees with POP. We found that the key SNP in the haplotype 9q21 associated with POP in our Russian population had the best RegulomeDB score 1a and two other SNPs category 1 were involved in SNP-SNP interaction associations. To the best of our knowledge, this is the first successful research which was performed utilizing RegulomeDB annotations a priori, for planning investigations. RegulomeDB annotations can be useful for designing an association study of the chromosome region.

## Supplementary Material

Table S1. Summary on primer sequences, amplicons, and PCR cycling conditions for individual SNPs analyzed in the study.
Table S2. Characteristics of the POP and control groups involved in the study.
Table S3. The distribution of genotypes among cases and controls.
Table S4. Linkage disequilibrium analysis of six SNPs in the region 9q21.
Table S5. Fst pairwise values between populations.

## Figures and Tables

**Table 1:** SNPs considered in this study.

rs	Chromosome region	Chromosome position assembly GRCh37.p13	Gene	Function	RegulomeDB score	Alleles	MAF (Allele)
rs1455311	4q21	79964587	*LINC01088 *	Intron	5	C:T	0.217 (C)
rs1036819	8q24	135611945	*ZFAT-AS1*, *ZFAT *	NearGene-3	4	A:C	0.128 (C)
rs4077632	9q21	81163473	—	—	No data	A:G	0.310 (G)
rs2807303	9q21	82187095	*TLE4 *	NearGene-5	2a	C:T	0.336 (T)
rs2777781	9q21	84215034	*TLE1 *	Intron	3a	A:T	0.309 (T)
rs11139451	9q21	84495608	—	—	5	C:T	0.221 (C)
rs12237222	9q21	85834743	*LOC102723989 *	Intron	1a	G:T	0.425 (G)
rs12551710	9q21	86088295	*FRMD3 *	Intron	1d	C:T	0.042 (T)
rs430794	9q22	93852815	—	—	5	A:C	0.272 (A)
rs8027714	15q11	24964597	—	—	6	A:G	0.040 (A)
rs1810636	20p13	2654925	—	—	4	G:T	0.332 (T)
rs2236479	21q22	46919132	*COL18A1 *	Intron	1f	A:G	0.339 (A)

**Table 2:** The distribution of RegulomeDB category 1a–1f SNPs in the whole human genome and in the region 9q21.

RegulomeDB category	Category description	Number of SNPs with RegulomeDB score 1a–1f	
		In the whole human genome	In the region 9q21
1a	eQTL + TF binding + matched TF motif + matched DNase footprint + DNase peak	352	1
1b	eQTL + TF binding + any motif + DNase footprint + DNase peak	2568	17
1c	eQTL + TF binding + matched TF motif + DNase peak	85	0
1d	eQTL + TF binding + any motif + DNase peak	1668	7
1e	eQTL + TF binding + matched TF motif	54	0
1f	eQTL + TF binding/DNase peak	34706	127

**Table 3:** The distribution of rs12237222 genotypes among cases and controls.

Genotypes	Control		POP		Crude* P* value, OR (95% CI)	Adjusted *P* value^a^OR, (95% CI)
	Number (%) *n* = 291	HWP	Number (%) *n* = 210	HWP		
rs12237222						
T/T	104 (35.7)	0.63	55 (26.2)	0.21	0.023 (dom) 1.57 (1.06–2.31)	0.12 (dom) 1.41 (0.91–2.18)
T/G	136 (46.7)		114 (54.3)			
G/G	51 (17.5)		41 (19.5)			

HWP: Hardy-Weinberg probability; OR: odds ratio; CI: confidence interval; dom: dominant model.

^a^Adjusted by age, body mass index (BMI), perineal trauma in childbirth, and vaginal parity. In multivariate analysis, there were 271 controls and 198 cases.

**Table 4:** The distribution of 9q21 haplotypes among cases and controls.

Number	rs4077632	rs2807303	rs2777781	rs11139451	rs12237222	rs12551710	Frequencies		*P* value	OR (95% CI)
							Controls	POP		
Six SNP-haplotype associations with response										
1	A	T	A	T	T	C	0.1334	0.0985	—	1.00
2	A	C	A	T	T	C	0.1185	0.0943	0.38	0.59 (0.19–1.90)
3	G	C	A	T	G	C	0.0436	0.0937	0.24	1.92 (0.65–5.71)
**4**	**A**	**C**	**A**	**T**	**G**	**C**	**0.059**	**0.1019**	**0.029**	**3.36 (1.13**–**9.95)**

Five SNP-haplotype associations with response										
1	A	C	A	T	—	C	0.1807	0.205	—	1.00
2	A	T	A	T	—	C	0.1662	0.1324	0.96	1.02 (0.48–2.16)
3	G	C	A	T	—	C	0.1146	0.1171	0.68	0.85 (0.39–1.86)
4	A	C	T	T	—	C	0.0949	0.0763	0.63	0.81 (0.34–1.92)

Two SNP-haplotype associations with response										
1	—	—	A	—	T	—	0.4581	0.3663	—	1.00
**2**	—	—	**A**	—	**G**	—	0.2782	0.3575	**0.0091**	**1.72 (1.15**–**2.59)**
3	—	—	T	—	T	—	0.1328	0.167	0.064	1.62 (0.97–2.70)
4	—	—	T	—	G	—	0.1309	0.1092	0.63	0.88 (0.53–1.47)

Only haplotypes with total frequencies ≥10% are considered. Analysis is adjusted by age, body mass index (BMI), peritoneal trauma in childbirth, and vaginal parity. Significant results are in bold.
