# Supplementary material for: Verification of the Chromosome Region 9q21 Association with Pelvic Organ Prolapse Using RegulomeDB Annotations
Source: Biomed Res Int. 2015 Aug 10;2015:837904. doi: 10.1155/2015/837904 (PMC4546950; doi:10.1155/2015/837904)
Supplement: Supplementary file 1 — Table S1. Summary on primer sequences, amplicons, and PCR cycling conditions for individual SNPs analyzed in the study. Table S2. Characteristics of the POP and control groups involved in the study. Table S3. The distribution of genotypes among cases and controls. Table S4. Linkage disequilibrium analysis of six SNPs in the region 9q21. Table S5. Fst pairwise values between populations. [file 837904.f1.pdf]

Verification of the chromosome region 9q21 association with pelvic organ prolapse using RegulomeDB annotations

Maryam B. Khadzhieva, Dmitry S. Kolobkov , Svetlana V. Kamoeva, Anastasia V. Ivanova, Serikbay K. Abilev, and Lyubov E. Salnikova

Supplementary Materials

SupplementaryTable 1. Summary on primer sequences[a], amplicons, and PCR cycling conditions for individual SNPs analyzed in the study

rs# of SNP	Primer sequences 5'-3', external forward/reverse, internal allele-specific forward/reverse	PCR[b]	Amplicon length (bp)
rs1455311	F aagtcctacccatcgcaggt	95ºC (15s)	303
	R ccggtaataatgggatgtgg	62ºC (15s)	
	F(t) ccatttctgaattacagagggt	72 ºC (20s)	150
	R(c) caggaaccttaatgaaaaatcaatag	32 ×	200
rs1036819	F aaagcccatgctctgaccta	95ºC (15s)	404
	Rttttagtctctgggagagaatgtc	60ºC (15s)	
	F(c)cactttttgctgtctgtagcataaac	72ºC (20s)	288
	R(a) ggttctgttttaattgaatgcctt	31 ×	165
rs4077632	F ggctagctcctgggaacaac	95 ºC (15s)	560
	R tgcaagctgtcatcagcata	60.6ºC(14s)	
	F(a) gagcaaggtacctttgcttga	72ºC(15s)	369
	R(g) cgataagaattagagaatcgc	31 ×	232
rs2807303	F cgtcccactccaaacttttt	95 ºC (15s)	344
	R gtggtgttcgcagagtttga	60 ºC (20s)	

	F(c) cttttaccacatctcatttccttatttc	72 °C (20s)	153
	R(t)gacaagaaaacaggaaggaagaa	31×	241
rs2777781	F tgacctaagttgataaatgaggaa	94 °C (15s)	343
	R tccctcttcaccttgtcagaa	62.3 °C (20s)	
	F(a) ggcatgaacaaggctccta	72 °C (22s)	227
	R(t) ctccatttgtatctttgtcctgtta	32 ×	159
rs11139451	F aaggaccattctaaagtttgtggac	95 °C (15s)	341
	R actgatgcaatagggacatgagata	61 °C (20s)	
	F(t) agggaagaaagctgtct	72 °C (20s)	151
	R(c) caagagttctgtgttttctg	15 ×	226
		95 °C (15s)	
		50.8 °C (20s)	
		72 °C (15s)	
		16 ×	
rs12237222	Fggatttaaccaggggccgttt	95 °C(15s)	370
	Rgcatccaagctccgcttcct	66°C(15s)	
	F(g)cttcttgtgggtttcccactgg	72 °C (15s)	149
	R(t)aaggacgaggctgctcca	30 ×	260
rs12551710	F aggactccagcccctagact	95 °C(15s)	334
	R cttggagtttacttgtggggaaa	63°C(15s)	
	F(t) tccagtcctccactcacctat	72 °C (30s)	150
	R(c)cacagaacctgctgtggaagg	31 ×	225
rs430794	F ggtagtaagcctgcactctgc	95 °C (15s)	332
	R caactgagtggggagcattt	65.3°C (14s)	
	F(c) catcagctttcagtgccacc	72 °C (14s)	216

	R(a) gcagaacatcaagcaagttacagt	29 ×	159
rs8027714	F ccagcatcttctctctttctagc	95 ºC (15s)	277
	R acacatatgggcaaatgcag	61.2 ºC (15s)	
	F(g) tcaaggaatcctaattcttttatttag	72 ºC (25s)	191
	R(a) ccagaactttgttccctatactattaat	32 ×	140
rs1810636	F ggtgacagatcagcaaccac	95 ºC (15s)	557
	R cttcaacccaggaggaagag	59 ºC (15s)	
	F(g) cctctaaacaattttttttaaagcg	72 ºC (25s)	236
	R(t) gacacactcaccaagcacata	30 ×	365
rs2236479	F gggctctagagggaacagtg	95 ºC (15s)	379
	R cctctgacctccctgtcg	59.6 ºC (20s)	
	F(a) cgtgtccttcagaacgaa	72 ºC (30s)	168
	R(g) ctgtcctgcatgtcatcc	31 ×	246

[a]The Primer3 algorithm was used to design external primer sequences.

[b]PCR conditions are provided as temperature (duration) of denaturation, annealing and extension phase, respectively, followed by the number of cycles for a given SNP. Each PCR reaction was finished by a final extension phase lasting 5 min.

Supplementary Table 2. Characteristics of the POP and control groups involved in the study

Characteristics	Control<br>N (%)<br>Mean ± SD	POP<br>N (%)<br>Mean ± SD	*P*[e]
Total number	292	210	
POP-Q stage			
Without POP	292 (100)	0 (0.00)	
Stage I	0 (0.00)	0 (0.00)	
Stage II	0 (0.00)	0 (0.00)	
Stage III	0 (0.00)	194 (92.38)	
Stage IV	0 (0.00)	16 (7.62)	
Age[a]	57.25 ± 12.70	57.65 ± 10.80	0.68
BMI (kg/m^2)[b]	27.46 ± 6.56	29.17 ± 5.85	**5.6×10^{-4}**
Vaginal parity[c]			
0	57 (20.73)	4 (1.96)	**0.014[f]**
1	112 (40.73)	79 (38.73)	
2	100 (36.36)	96 (47.06)	
≥3	6 (2.18)	25 (12.25)	
Perineal trauma in childbirth (episiotomy and spontaneous perineal laceration)[d]	93 (32.75)	107 (52.45)	**1.7×10^{-5}**

Data are missing for: [a]8 controls, 6 POP patients; [b]11 controls, 12 POP patients; [c]17 controls, 6 POP patients; [d]91 controls, 51 POP patients.

[e]Significant results are in bold.

[f]We compared two groups: 0-1 vaginal birth vs. ≥ 2 vaginal birth.

Supplementary Table 3. The distribution of genotypes among cases and controls

SNPs and genotypes		Control		POP		Crude P– value, OR, (95% CI)	Adjusted P– value[a], OR, (95% CI)
		Number (%)	HWP	Number (%)	HWP		
rs1455311		n=287		n=210			
	T/T	187 (65.2)		146 (69.5)		0.24 (add)	0.10 (add)
	T/C	91 (31.7)	0.70	60 (28.6)	0.61	0.82	0.72
	C/C	9 (3.1)		4 (1.9)		(0.58 – 1.15)	0.48 – 1.07
rs1036819		n=290		n=211			
	A/A	232 (80.0)		156 (74.3)		0.13 (dom)	0.11 (dom)
	A/C	54 (18.6)	0.55	50 (23.8)	1.00	1.38	1.49
	C/C	4 (1.4)		4 (1.9)		(0.91 – 2.11)	0.92 – 2.41
rs4077632		n=291		n=209			
	A/A	128 (44.0)		79 (37.8)		0.094 (add)	0.22 (add)
	A/G	134 (46.0)	0.51	101 (48.3)	0.77	1.26	1.20

	G/G	29 (10.0)		29 (13.9)		(0.96 – 1.64)	(0.89 – 1.62)
rs2807303		n=285		n=210			
	C/C	102 (35.8)		80 (38.1)		0.19 (rec)	0.38 (rec)
	C/T	142 (49.8)	0.53	108 (51.4)	0.13	0.79	0.76
	T/T	41 (14.4)		22 (10.5)		(0.40 – 1.21)	(0.41 – 1.40)
rs2777781		n=292		n=210			
	A/A	154 (52.7)		111 (52.9)		0.25 (rec)	0.56 (rec)
	A/T	122 (41.8)	0.23	82 (39.0)	0.73	1.52	1.25
	T/T	16 (5.5)		17 (8.1)		(0.75 – 3.08)	(0.59 – 2.67)
rs11139451		n=291		n=210			
	T/T	185 (63.6)		131 (62.4)		0.78 (dom)	0.74 (dom)
	T/C	88 (30.2)	0.11	70 (33.3)	1.00	1.05	1.07
	C/C	18 (6.2)		9 (4.3)		(0.73 – 1.52)	(0.71 – 1.62)
rs12551710		n=291		n=210			
	C/C	233 (80.1)		171 (81.4)		0.58 (add)	0.70 (add)
	C/T	56 (19.2)	0.75	39 (18.6)	0.23	0.88	0.91

	T/T	2 (0.7)		0 (0.0)		(0.57 – 1.37)	(0.56 – 1.47)
rs430794		n=289		n=210			
	C/C	148 (51.2)		122 (58.1)		0.13 (dom)	0.11 (dom)
	C/A	113 (39.1)	0.39	72 (34.3)	0.27	0.76	0.72
	A/A	28 (9.7)		16 (7.6)		(0.53 – 1.08)	(0.49 – 1.08)
rs8027714		n=290		n=210			
	G/G	278 (95.9)		197 (93.8)		0.30 (dom)	0.27 (dom)
	G/A	12 (4.1)	1.00	13 (6.2)	1.00	1.53	1.66
	A/A	0 (0.0)		0 (0.0)		(0.68 – 3.42)	(0.67 – 4.09)
rs1810636		n=290		n=210			
	G/G	119 (41.0)		101 (48.1)		0.12 (dom)	0.25 (dom)
	G/T	144 (49.7)	0.09	91 (43.3)	0.75	0.75	0.79
	T/T	27 (9.3)		18 (8.6)		(0.53 – 1.07)	(0.53 – 1.18)
rs2236479		n=287		n=207			
	G/G	131 (45.6)		81 (39.1)		0.14 (add)	0.097 (add)
	A/G	120 (41.8)	0.29	94 (45.4)	0.66	1.22	1.28

A/A	36 (12.5)	32 (15.5)	(0.94 – 1.57)	(0.96 – 1.71)

HWP, Hardy– Weinberg probability; OR, odds ratio; CI, confidence interval; rec, recessive model; dom, dominant model; add, additive model.

[a]Adjusted by age, body mass index (BMI), perineal trauma in childbirth and vaginal parity. In the multivariate analysis, the number of cases and controls may be less than in the crude analysis since some subjects had insufficient data on covariates.

Supplementary Table 4. Linkage disequilibrium analysis of six SNPs in the region 9q21

D'<br>*P*– value	rs11139451	rs12237222	rs12551710	rs2777781	rs2807303
rs4077632	0.149	0.034	0.323	0.009	0.022
	0.073	0.367	4.17×10^{-6}	0.895	0.515
rs11139451		0.257	0.129	0.236	0.0007
		1.45×10^{-6}	0.009	0.019	0.988
rs12237222			0.266	0.052	0.098
			0.001	0.252	0.035
rs12551710				0.043	0.373
				0.456	0.002
rs2777781					0.177
					0.008

D' and *P* – value for linkage are given for every pair of SNPs.

Supplementary Table 5. Fst pairwise values between populations

Pops	CHD	GIH	JPT	TSI	YRI	RUS	MEX	LWK
CEU	0.1108	0.0324	0.0753	0	0.1574	0	0.0236	0.1654
CHD		0.0492	0.0148	0.1302	0.1951	0.1104	0.0712	0.1822
GIH			0.029	0.0435	0.1548	0.0297	0.0159	0.1341
JPT				0.079	0.1749	0.0689	0.0499	0.1562
TSI					0.1681	0.0017	0.0281	0.1638
YRI						0.1482	0.1148	0.0093
RUS							0.0256	0.1575
MEX								0.101
